# Prescribing Pattern and Safety of Immunosuppressants in Renal Transplant Patients: An Observational Study

**DOI:** 10.7759/cureus.46200

**Published:** 2023-09-29

**Authors:** Lavanya Ragavanandam, Kulur M Sudha, Sankalp Yadav

**Affiliations:** 1 Pharmacology, Faculty of Medicine, Sri Lalithambigai Medical College and Hospital, Dr. M.G.R. Educational and Research Institute, Chennai, IND; 2 Pharmacology, Madras Medical College, Chennai, IND; 3 Medicine, Shri Madan Lal Khurana Chest Clinic, New Delhi, IND

**Keywords:** adverse drug reactions (adr), renal transplantation, immunosuppressant, prescribing pattern, induction therapy, maintenance therapy

## Abstract

Background: Renal transplantation is a life-saving procedure and contributes to a better quality of life in patients with end-stage renal disease. The discovery and use of immunosuppressants to prevent and treat allograft rejection are responsible for the improved outcome after the transplant. Long-term usage of these drugs warrants special monitoring and follow-up of the adverse drug reactions developed during the post-transplant period. This study analyzes the prescribing pattern and severity and outcome of adverse drug reactions of immunosuppressants in renal transplant patients.

Materials and methods: A cross-sectional, observational study was done in patients more than 18 years of age who have undergone renal transplantation and receiving immunosuppressants in the Department of Nephrology in a tertiary care hospital.

Results: During the two-month study period, 150 post-transplant patients were screened for adverse drug reactions, and the prescription pattern was also studied. Immunosuppressive therapy was given as induction and maintenance therapy. The short-term induction therapy regimen was based on the type of donor (injection basiliximab or anti-thymocyte globulin). The long-term maintenance therapy comprises triple therapy of tacrolimus, mycophenolate mofetil, and prednisolone in 102 (68%) patients and cyclosporine, mycophenolate mofetil, prednisolone in 37 (25%) patients. A total of 116 adverse drug reactions were reported in 82 patients. The pattern of the adverse drug reactions showed urinary tract infections in 26 (17.3%) patients on tacrolimus-based regimen and hypertension in 20 (13.3%) patients on tacrolimus and cyclosporine-based regimen. Causality assessment using the World Health Organization causality assessment scale showed that the observed reactions were of probable 42 (36%) and possible 74 (64%) categories.

Conclusions: Long-term intake of immunosuppressive drugs is essential to improve the quality of life in renal transplant individuals and it is essential to monitor adverse drug reactions of these drugs through vigilant self-reporting and pharmacovigilance practices.

## Introduction

Renal transplantation (RT) provides life-saving treatment for patients with end-stage renal disease (ESRD) [1]. Marked improvements in early graft survival and long-term graft function by immunosuppressive agents have made kidney transplantation a better alternative to dialysis [2]. As per 2015 statistics, the prevalence of ESRD requiring transplantation in India is estimated to be between 151 and 232 per million population. Approximately 220,000 people require kidney transplantation in India, of which 7,500 transplants are done. Of these, 90% come from living donors and 10% from deceased donors [3].

The total success of RT is hugely dependent on the proper use of immunosuppressive therapy. Kidney transplantation and associated immunosuppressive therapy have greatly evolved [4]. From the early 1960s, azathioprine and steroids were the backbones of immunosuppression in RT [5]. With the introduction of calcineurin inhibitors (cyclosporine, tacrolimus), there was a significant reduction in rejection rates and an increase in graft survival rates [6]. However, no particular regimen has proved to be superior to another.

Advances in immunosuppressive therapy and refinement in surgical techniques have allowed RT to evolve into an optimal therapy for ESRD. Management of ESRD not only includes successful kidney transplantation but also involves treatment of post-transplant complications such as graft failure, and acute and chronic rejection. Immunosuppressive therapies in the form of induction and maintenance therapy are given to renal transplant patients which aids in the reduction of post-transplant complications.

Induction therapy aims to provide a high level of immunosuppression, to protect the graft from the host’s early immune response [1]. Biological immunosuppressants like basiliximab and anti-thymocyte globulin (ATG) are used in induction and rescue therapies [5]. The goal of maintenance therapy is to provide a low or moderate level of immunosuppression to save the graft from rejection [1]. Triple therapy comprising a calcineurin inhibitor (cyclosporine/tacrolimus), glucocorticoids, and anti-proliferative drug (mycophenolate mofetil (MMF)/azathioprine) are commonly employed [6]. When rejection is suspected, a graft biopsy is done for confirmation, and empirical treatment with high doses of corticosteroids mainly 500-1,000 mg of methylprednisolone intravenous (IV) is given for three days [1].

Conventional immunosuppressive protocols consist of an antibody induction (basiliximab, ATG) and triple therapy for maintenance (calcineurin inhibitors + steroids + anti-proliferative drug). With conventional protocols, most programs can achieve 90%-95% graft survival with an acute rejection rate of 10%-20% [5]. Apart from conventional immunosuppressive protocol, each transplant center has its prescribing pattern (protocol). Protocols should be regarded as guides of therapy that may require modification from patient to patient depending upon the patient’s response and tolerance to the drug.

Chronic kidney disease is associated with alteration in the mechanisms involved in the intestinal transport of drugs, thereby it affects the bioavailability of the immunosuppressants, leading to an increased risk of development of adverse drug reactions (ADRs) [7]. ADR is defined by the World Health Organization (WHO) as “a response which is noxious and unintended and which occurs at doses normally used in humans for the prophylaxis, diagnosis or therapy of disease, or for the modification of physiological function” [8].

ADRs can affect the compliance and patient’s comfort level with the drug. Generally, the multidrug regimen is followed which allows the use of low doses of individual agents, thus reducing the severity of dose-related adverse effects [1]. The lifelong requirement of immunosuppressants after transplant is associated with several ADRs and periodic monitoring is required to minimize the risk and frequency of adverse effects.

The study of prescription patterns explains the extent and profile of drug use, trends, and compliance with the standard treatment guidelines. The inappropriate use of medicines results in the wastage of resources and health hazards. ADRs are one of the prime concerns to deal with while treating patients on Immunosuppressive drugs, as they require life-long treatment. Assessment of the benefits, harm, and risks of medicines and improvement of public health and safety regarding the use of medicines are the prime aims of the field of pharmacovigilance [9]. Due to chronic administration of immunosuppressants, renal transplant recipients are more likely to develop ADRs, and this serves as a major threat to their quality of life.

In several studies, it has been noticed that there are more frequent changes in the immunosuppressant regimen within a year of a transplant due to the development of adverse reactions [6]. The actual prescription pattern of immunosuppressants in kidney transplantation remains unclear. Hence, the main objective of this study is to evaluate the prescribing pattern and ADRs occurring in renal transplant patients receiving immunosuppressants in a tertiary care hospital. The severity and the outcome of each of the ADRs occurring in the study participants are assessed, analyzed, and managed accordingly. Prescription pattern monitoring and spontaneous ADR reporting by healthcare professionals help to identify, quantify, and document drug-related problems. It also contributes to reducing drug-related problems and increases knowledge and understanding of the mechanisms underlying drug-induced reactions [9].

## Materials and methods

After obtaining institutional ethical clearance (ECR/270/Inst./TN/2017), a total of 150 patients who had undergone RT and received immunosuppressants attending the outpatient department of Nephrology in a tertiary care hospital were recruited. The post-renal transplant patients on immunosuppressive therapy, who were 18-60 years of age and willing to participate were included and the rest of them were excluded from the study. ADRs due to non-compliance or overdose were also excluded.

This cross-sectional observational study was done for a period of two months (November 2017-December 2017) in the Nephrology outpatient department (OPD), Rajiv Gandhi Government General Hospital, in collaboration with the Institute of Pharmacology, Madras Medical College, Chennai, India. RT patients receiving immunosuppressant treatment at Nephrology OPD were explained the study purpose in their local language. Written informed consent was obtained from those who were willing to participate in this study. The following parameters were recorded from the patient’s medical record: patient’s demographic details, type of donor, co-morbid medical conditions, indications for kidney transplantation, induction, and maintenance therapy regimen, change in the regimen and its reason, drug used in the treatment of co-morbid conditions and renal function tests.

The patients were interviewed about the occurrence of ADRs in the presence of healthcare professionals, i.e., doctors and nurses. ADRs were collected by filling in the ADR form either by the patient or by the treating health officials. The collected ADR forms were analyzed and the ADR pattern stating the incidence, severity, and outcomes were tabulated. Severity and causality assessment of the ADR was done by establishing a temporal association of drug use with ADR using the Modified Hartwig severity assessment scale and WHO causality assessment scale, respectively. Treatment done for the ADRs was also recorded. Immunosuppressive therapy for renal transplant patients recruited for the study was given according to the department protocol.

Induction regimen

Live Donors

Injection basiliximab 20mg IV (2 doses- 1st dose at the time of surgery and 2nd dose on the fourth post-operative day) + Injection methylprednisolone 1000mg IV stat dose (at the time of surgery).

Deceased Donors

Injection ATG 1.5mg/kg IV stat dose + Injection methylprednisolone 1,000mg IV stat dose (at the time of surgery).

Maintenance regimen

Irrespective of the donor status following induction therapy, they were given the following maintenance therapy.

Preferred Regimen

Tablet tacrolimus 1-3mg twice daily (BD) + Tablet MMF 500mg three times a day (TDS) + Tablet prednisolone 30mg once a day (OD)

Alternative Regimen

(a) Tablet cyclosporine 100-175mg BD + Tablet MMF 500mg TDS + Tablet prednisolone 30mg OD

(b) Tablet tacrolimus 1-3mg BD + Tablet azathioprine 500mg BD + Tablet prednisolone 30mg OD

(c) Tablet cyclosporine 100-175mg BD+ Tablet azathioprine 500mg BD + Tablet prednisolone 30mg OD

Drug dosage was adjusted according to the trough level of the drug and patient response. Normal trough levels of tacrolimus were 5-20 ng/L and that of cyclosporine was 150-400 ng/mL. An alternative regimen was followed in case of the development of a serious adverse reaction that warrants discontinuation.

Statistical analysis

Descriptive statistics were reported as mean (SD) for continuous variables and frequencies (percentage) for categorical variables. The chi-square test was used to find the association between categorical variables. Multivariate logistic regression was used to find the association between the dependent and independent variables. Data was statistically evaluated with IBM SPSS Statistics for Windows, Version 26.0 (IBM Corp., Armonk, NY).

## Results

Among 150 post-renal transplant patients enrolled in the study, 99 were males and 51 were females. Males were commonly affected. Their mean age was 36 years. Type 2 diabetes mellitus (T2DM) was the commonly seen co-morbid condition in post-renal transplant study patients. Other associated concomitant illnesses in the patients are shown in Table 1.

**Table 1:** Co-morbidities in renal transplant patients

Co-morbidities	Number of patients	Percentage of patients
Type-2 diabetes mellitus only	71	56%
Hypertension only	34	22%
Diabetes and hypertension	22	12%
Coronary artery disease	5	2%
Total	92	61%

Immunosuppressive agents were prescribed per the department protocol concerning the type of donor (live/deceased) and type of therapy (induction/maintenance). Induction therapy regimens were received by the patients based on the type of donor as tabulated in Table 2.

**Table 2:** Prescribing pattern of induction therapy regimen

Donor	Induction regimen	Number of patients	Percentage of patients
Live	Injection basiliximab + Injection methylprednisolone	91	60.6%
Deceased	Injection anti-thymocyte globulin + Injection methylprednisolone	59	39.3%

**Figure 1:**
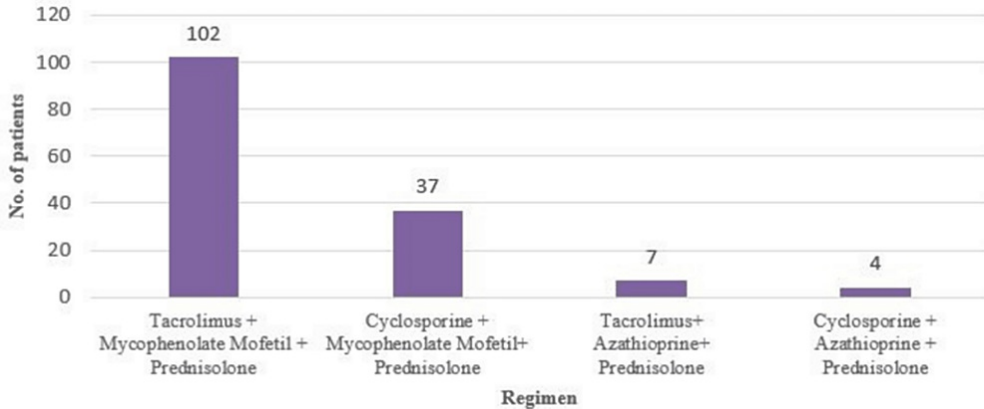
Prescription pattern of maintenance therapy - triple therapy regimen No. - Number

Following induction therapy, the study patients were given maintenance therapy consisting of a combination of three classes of immunosuppressive agents (calcineurin inhibitor + anti-proliferative agent + corticosteroids) as illustrated in Figure 1.

The majority of them were given tacrolimus and MMF-based regimens for maintenance. The study population was also prescribed other classes of drugs in addition to maintenance therapy. All the patients were receiving supplements like vitamins and minerals. The other concomitant medications include insulin, oral hypoglycemic drugs, anti-hypertensives, hypolipidemic, co-trimoxazole, valganciclovir, and anti-ulcer drugs.

A total of 116 ADRs were reported in 82 patients during the study. Urinary tract infections and hypertension (HT) were the commonest adverse events reported (Table 3). The WHO causality assessment determined that 64% of the patients who developed ADRs belonged to the “possible” category and 36% of the patients in the “probable” category.

**Table 3:** Pattern of adverse drug reactions in renal transplant patients on immunosuppressive drugs CMV - Cytomegalovirus, MMF - Mycophenolate mofetil

Adverse reactions	Number of patients	Percentage of patients	Suspected drug
Urinary tract infections	26	17.30%	Tacrolimus
Hypertension	20	13.30%	Cyclosporine, tacrolimus
Tremors	18	12%	Tacrolimus
Nausea, vomiting	15	10%	MMF
Headache	13	8.60%	Tacrolimus
Elevated urea, creatinine	6	4%	Tacrolimus, cyclosporine
Elevated liver enzymes	6	4%	Tacrolimus
CMV diarrhea	5	3.30%	Mycophenolate mofetil
Granulocytopenia	2	1.30%	Valganciclovir
Seizures	1	0.60%	Tacrolimus
Gingival hyperplasia	1	0.60%	Cyclosporine, tacrolimus
BK viral infection	1	0.60%	Cyclosporine, MMF
Cushingoid features	1	0.60%	Prednisolone
Osteoporosis	1	0.60%	Prednisolone
Total	116		

The severity assessment was also done using the Modified Hartwig Siegel scale and the majority of them were in the “mild” category and the severity of each of the ADRs is given in Table 4. Statistically hypertensives were common among grade 2. Similarly, granulocytopenia seizures and gingival hyperplasia were more among grade 2 and grade 3.

**Table 4:** Severity assessment of adverse drug reactions according to modified Hartwig Siegel scale CMV - Cytomegalovirus

Adverse Drug Reactions	No. of patients	Grade 1 (Mild)	Grade 2 (Moderate)	Grade 3 (Severe)	X^2^, (df), P-value
Urinary tract infections	26	17	9	0	0.22 (2), 0.89
Hypertension	20	7	13	0	8.28 (2), 0.02
Tremors	18	15	3	0	3.78 (2), 0.15
Nausea, Vomiting	15	11	4	0	0.82 (2), 0.66
Headache	13	9	4	0	0.36 (2), 0.83
Elevated urea, creatinine	6	4	2	0	1.32 (2), 0.52
Elevated liver enzymes	6	5	1	0	2.30 (2), 0.32
CMV diarrhea	5	2	2	1	3.37 (2), 0.18
Granulocytopenia	2	0	1	1	6.46 (2), 0.04
Seizures	1	0	0	1	6.52 (2), 0.04
Gingival hyperplasia	1	0	0	1	6.52 (2), 0.04
BK viral infection	1	0	1	0	5.20 (2), 0.07
Cushingoid features	1	0	1	0	5.20 (2), 0.07
Osteoporosis	1	0	0	1	6.52 (2), 0.04
TOTAL	116	70 (61%)	41 (35%)	5 (4%)	

Multivariable regression analysis of ADRs shows urinary tract infection were 1.14 times more among grade 1, tremors 3.45 times more among grade 1, nausea and vomiting 1.72 times more among grade 1 and elevated liver enzymes were 3.07 times more among grade 1. But these associations were not found to be statistically significant (Table 5).

**Table 5:** Multivariable regression analysis of adverse drug reactions ORA - Odds ratio, CI - Confidence interval, SE - Standard error, CMV - Cytomegalovirus

Adverse Drug Reactions	Number of patients	Grade 1 (Mild)	Grade 2 and Grade 3 (Moderate/ Severe)	P	B	SE	ORA, 95% CI
Urinary tract infections	26	17	9	0.38	0.122	0.219	1.14 (0.45 to 2.86)
Hypertension	20	7	13	0.001	-1.47	0.271	0.23 (0.08 to 0.66)
Tremors	18	15	3	0.03	1.23	0.445	3.45 (0.93 to 12.76)
Nausea, vomiting	15	11	4	0.19	0.54	0.38	1.72 (0.51 to 5.82)
Headache	13	9	4	0.31	1.35	0.404	1.36 (0.39 to 4.74)
Elevated urea, creatinine	6	4	2	0.42	0.17	0.79	1.18 (0.21 to 6.75)
Elevated liver enzymes	6	5	1	0.16	1.12	1.24	3.07 (0.34 to 27.29)
CMV diarrhea	5	2	3	0.29	-0.66	0.56	0.57 (0.07 to 4.23)
Granulocytopenia	2	0	3	NA	NA	NA	NA
Seizures	1	0	1	NA	NA	NA	NA
Gingival hyperplasia	1	0	1	NA	NA	NA	NA
BK viral infection	1	0	1	NA	NA	NA	NA
Cushingoid features	1	0	1	NA	NA	NA	NA
Osteoporosis	1	0	1	NA	NA	NA	NA
TOTAL	116	70 (61%)	41 (35%)				

**Figure 2:**
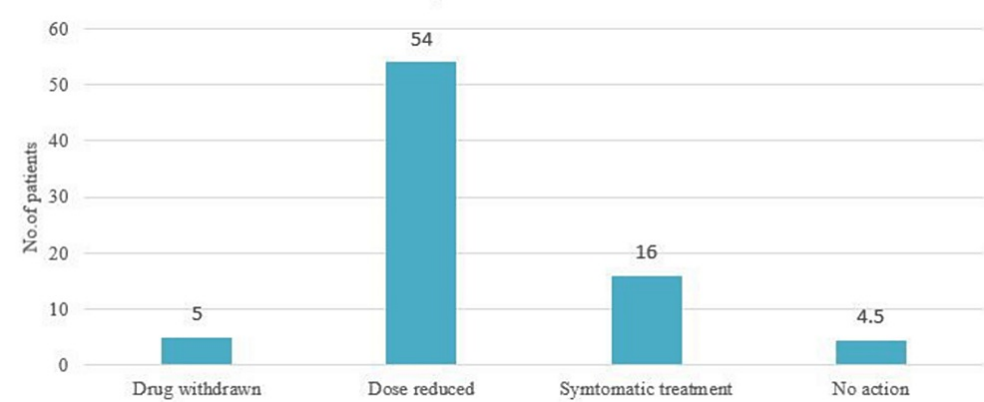
Action taken for the adverse drug reactions No. - Number

The ADRs were documented, categorized, and treated. Dose reduction of the causative immunosuppressive drug forms the major action taken in the management of ADRs due to the maintenance therapy drugs in post-renal transplant patients (Figure 2).

## Discussion

In this study, among 150 post-renal transplant patients, 66% were males and 34% were females. The prevalence was two times higher in males than females. 74% of study patients belong to the age group 40-49 years. 61% of the renal transplant patients had concomitant illnesses like T2DM, HT, T2DM + HT, and coronary artery disease.

The prescription pattern of Immunosuppressive drugs was given as per the department protocol. Depending upon the type of donor, induction therapy was given. 61% of live donors received an injection of basiliximab + injection of methylprednisolone and 39% of deceased donors received an injection of ATG + injection of methylprednisolone at the time of surgery. None of the patients developed ADRs prior to the induction therapy.

One hundred and two patients (68%) were on tacrolimus + MMF + prednisolone during the maintenance phase. Only 4% were on cyclosporine and azathioprine-based regimen. Tablet prednisolone was started for all patients at a dose of 60mg and slowly tapered up to 30mg by reducing 10mg every week. Vitamins and minerals were co-prescribed for almost all patients. Out of the other classes of drugs prescribed, 64 patients received the tablet valganciclovir 450mg OD for three months and the tablet cotrimoxazole (80/400mg) OD as prophylaxis against opportunistic infections due to prolonged immunosuppression. Antiulcer drugs like omeprazole and ranitidine were prescribed in 112 patients to reduce the gastric upset caused by prednisolone. Oral hypoglycemic drugs like tablet metformin, and anti-hypertensives like tablet atenolol, tablet amlodipine, and tablet prazosin, tablet atorvastatin were the other classes of drugs co-prescribed in these patients.

A total of 116 ADRs were reported in 82 patients during the study. Urinary tract infections due to tacrolimus were the most common ADR reported by patients and constitute 17.3% of all ADRs. The second common ADR was HT (13.3%) due to tacrolimus and cyclosporine.

After the causality assessment, 42 ADRs were “Probable,” and 74 were “Possible” ADRs. According to the modified Hartwig Seigel Scale, severity assessment of ADRs showed 61% of ADRs belonging to the “Mild” category. In the management of ADRs, a dose of the drug was reduced in the majority of the cases. In 54 cases out of 82, the drug was withdrawn in five cases only. This shows that Immunosuppressive drugs are an integral part of post-transplant therapy required to prevent rejection, so they are withdrawn when the risk seriously outweighs the benefit, or the patient is intolerant to the drug. The most serious adverse effects like seizures, granulocytopenia, cytomegalovirus (CMV) diarrhea (recurrent), and BK (polyomavirus) virus-induced nephropathy require withdrawal of suspected drugs.

Both serious and non-serious ADRs were noted during the maintenance phase. Prevalence of ADRs was categorized under the “Mild” category in this study, which reflects early detection of adverse reactions with the help of clinical presentation and drug trough levels and efficient management. Tacrolimus and cyclosporine-induced urinary tract infections and HT were predominantly seen. This correlated with increased blood levels of these drugs and warranted dose reduction of the above drugs [10]. One rare case of tacrolimus-induced isolated seizures was reported and the regimen was changed to cyclosporine-based triple therapy for that patient.

Ong et al. [11] conducted a single-center retrospective study comparing the impact of tacrolimus against cyclosporine on post-transplant outcomes in the kidney transplant population. In this study, they found comparable short-term post-transplant outcomes between cyclosporine and tacrolimus. Tacrolimus appears more tolerable but may be associated with infection risks.

The incidence rate of patients affected with ADRs was found to be 54.6%, which is similar to the studies by Moradi et al. and Bril et al. which show 55.5% and 54.42%, respectively [12,13]. Calcineurin inhibitors (tacrolimus, cyclosporine) are drugs with nephrotoxic potential and in this study, 6% of the patients had nephrotoxicity related to either cyclosporine or tacrolimus, a study from Wagle Shukla et al. supports this statement [14].

In this study, a rare case of tacrolimus-induced isolated seizures (one patient) was encountered. A spectrum of neurologic complications depending upon dose and blood levels are more common with tacrolimus but the complications are less severe in kidney transplants. Although tacrolimus-induced coarse tremors, headache, and insomnia are commonly observed, isolated seizures occur very rarely [14]. This patient was on tacrolimus + MMF + prednisolone regiment for the past two years. He developed two consecutive episodes of seizures. Tacrolimus's blood level was 32ng/mL, which was higher than the normal value (5-20ng/ml), so his regimen was changed to cyclosporine + MMF + prednisolone. He was started on the tablet phenytoin 100mg TDS for three months. His ADR was classified under the “Severe” category and the causality assessment was “Probable.”

Even though the patient was detected the earliest and symptomatic treatment and change of regimen was done, it is essential to establish standardized approaches and spontaneous reporting of ADRs by healthcare professionals to improve the quality of the healthcare system and of the patient. This study provides generalized and preliminary information about the prescription pattern and the type and frequency of ADRs associated with immunosuppressive drugs in renal transplant recipients. However, it has its limitations since it is a cross-sectional study done only for two months, all the patients with ADRs could not be followed up further, so the chronic effects of Immunosuppression were not documented well.

## Conclusions

Immunosuppressive drugs act by suppressing the immune system which itself contributes to the development of adverse reactions. The majority of the patients in this study were on triple therapy of tacrolimus/MMF/prednisolone combination. Induction therapy given based on donor status at the time of surgery was uneventful. Immunosuppressive drugs which play a key role in the success of RT, are also associated with several ADRs, which may affect the compliance and the quality of life of the patient. So, it is essential to monitor the effectiveness and safety of these drugs through prescription patterns ADR monitoring studies, and pharmacovigilance practices. This can be done by spontaneous and voluntary reporting of ADR by healthcare professionals and the patient themselves. Thus, special monitoring and periodic follow-up of patients are required to reduce drug-related health hazards.
